# Urban Aerosol Particulate Matter Promotes Necrosis and Autophagy via Reactive Oxygen Species-Mediated Cellular Disorders that Are Accompanied by Cell Cycle Arrest in Retinal Pigment Epithelial Cells

**DOI:** 10.3390/antiox10020149

**Published:** 2021-01-20

**Authors:** Hyesook Lee, Da Hye Kim, Jeong-Hwan Kim, Seh-Kwang Park, Ji-Won Jeong, Mi-Young Kim, Seok-Ho Hong, Kyoung Seob Song, Gi-Young Kim, Jin Won Hyun, Yung Hyun Choi

**Affiliations:** 1 Anti-Aging Research Center, Dong-eui University, Busan 47340, Korea; 14983@deu.ac.kr; 2 Department of Biochemistry, Dong-eui University College of Korean Medicine, Busan 47227, Korea; 3 Research and Development Department, BGN CARE Co., Ltd., Busan 47195, Korea; genebio97@bgncare.com (J.-H.K.); jung@bgncare.com (M.-Y.K.); 4 BGN Eye Clinic, Seoul 05551, Korea; psk11@bgncare.com; 5 BGN Eye Clinic, Busan 47195, Korea; jjw22@bgncare.com; 6 Department of Internal Medicine, School of Medicine, Kangwon National University, Chuncheon 24341, Korea; shhong@kangwon.ac.kr; 7 Department of Cell Biology and Biophysics, Kosin University College of Medicine, Busan 49267, Korea; kssong@kosin.ac.kr; 8 Department of Marine Life Sciences, Jeju National University, Jeju 63243, Korea; immunkim@jejunu.ac.kr; 9 Jeju National University School of Medicine and Jeju Research Center for Natural Medicine, Jeju 63243, Korea; jinwonh@jejunu.ac.kr

**Keywords:** mitophagy, necrosis, reactive oxygen species (ROS), retinal pigment epithelial (RPE) cells, urban aerosol particulate matter (UPM)

## Abstract

Urban particulate matter (UPM) is recognized as a grave public health problem worldwide. Although a few studies have linked UPM to ocular surface diseases, few studies have reported on retinal dysfunction. Thus, the aim of the present study was to evaluate the influence of UPM on the retina and identify the main mechanism of UPM toxicity. In this study, we found that UPM significantly induced cytotoxicity with morphological changes in ARPE-19 human retinal pigment epithelial (RPE) cells and increased necrosis and autophagy but not apoptosis. Furthermore, UPM significantly increased G2/M arrest and simultaneously induced alterations in cell cycle regulators. In addition, DNA damage and mitochondrial dysfunction were remarkably enhanced by UPM. However, the pretreatment with the potent reactive oxygen species (ROS) scavenger N-acetyl-L-cysteine (NAC) effectively suppressed UPM-mediated cytotoxicity, necrosis, autophagy, and cell cycle arrest. Moreover, NAC markedly restored UPM-induced DNA damage and mitochondrial dysfunction. Meanwhile, UPM increased the expression of mitophagy-regulated proteins, but NAC had no effect on mitophagy. Taken together, although further studies are needed to identify the role of mitophagy in UPM-induced RPE injury, the present study provides the first evidence that ROS-mediated cellular damage through necrosis and autophagy is one of the mechanisms of UPM-induced retinal disorders.

## 1. Introduction

Across Asia, urban air pollution has become part of the daily existence of citizens, and it is recognized as a grave public health problem [1,2]. Accumulated evidence has demonstrated that a high proportion of air pollutants adversely affect health and socioeconomic perspectives, such as an increase in medical costs and a reduction in productivity, for citizens living in urban areas of Europe [3]. Recently, the World Health Organization (WHO) cited that air pollutants are a major environmental cause of premature mortality [4]. Among urban air pollutants, urban particulate matter (UPM) contains various toxic substances, such as metals, biological contaminants, inorganic elements, and elemental and organ carbon [5]. Significantly, fine particulate matter (PM_2.5_) can remain in the atmosphere for over several hours to weeks, deeply penetrating into the major organs through breathing and skin [6,7]. In this regard, the latest epidemiological and toxicological studies have demonstrated that UPM results in negative biological effects on the respiratory tract [8], cardiovascular systems [9], immune systems [10], nervous systems [11], and skin [12].

Importantly, there has been increasing interest in the harmful effect of UPM on visual systems, which is an organ directly exposed to urban air pollutants [13,14,15]. In addition, some nonclinical studies have demonstrated that exposure to UPM leads to the apoptosis and inflammation of the corneal and conjunctival epithelium in vitro and in vivo [16,17]. More notably, recent studies have shown that the inner part of the eye may also be affected by urban air pollutants, because air pollution leads to systemic disease [18,19]. A few epidemiological studies have reported that urban air pollutants induce retinal disorders and result in increasing prevalence rates of retinal disorders including age-related macular degeneration (AMD), diabetic retinopathy, and sclerosis of the retina [19,20,21,22]. One animal study demonstrated that PM induces edema in retina [23]. More recently, Tien et al. [24] published results showing that perfluorooctanoic acid contained in PM induces oxidative stress and inflammation in the retinal pigment epithelium (RPE) and acts as a risk factor for AMD. In 2020, another study suggested that glial fibrillary acidic protein (GFAP), a hallmark of glial activation in accordance with neural damage, is markedly expressed by long-term exposure to UPM [16]. Although a few epidemiological studies have reported that UPM causes retinal dysfunction and increases the prevalence of retinal disorders, studies of the underlying mechanism are extremely limited. In this regard, our previous report demonstrated that diesel PM promotes migration and cellular dysfunction of RPE through reactive oxygen species (ROS) production, consequently inducing retinal dysfunction [25]. Our previous report is the first to establish that epithelial–mesenchymal transition (EMT) is the pathological mechanism of retinal exposure to PM. Nevertheless, the main pathological mechanism of retinal dysfunction exposure to UPM is still unknown. Therefore, to evaluate the influence of UPM on the retina, we investigated the cytotoxic effect of UPM in ARPE-19 human retinal pigment epithelial cells and identified the main mechanism of UPM cytotoxicity.

## 2. Materials and Methods

### 2.1. UPM Preparation

Urban aerosols (certified reference material No. 28) were obtained from the National Institute for Environmental Studies (NIES, Ibaraki, Japan) [26]. UPM was dissolved in a culture medium.

### 2.2. Cell Culture and Treatment

The human RPE cell line ARPE-19 was obtained from the American Type Culture Collection (Manassas, MD, USA). The cells were maintained in Dulbecco’s modified Eagle’s medium: nutrient mixture F-12 (Invitrogen-Gibco, Carlsbad, CA, USA) supplemented with 10% fetal bovine serum at 37 °C in a 5% CO_2_ incubator. When the cells were approximately 80% confluent, the cells were pretreated with or without 5 mM N-acetylcysteine (NAC, Sigma-Aldrich Chemical Co., St. Louis, MO, USA) or 200 μM necrostatin-1 (Sigma-Aldrich Chemical Co.). After 1 h, the cells were incubated with a desired concentration of UPM for 24 h.

### 2.3. Cytotoxicity Assay

Cytotoxicity was assessed using the CCK-8 assay kit (Abcam Inc., Cambridge, UK) to evaluate cytotoxicity according to the manufacturer’s instructions. The CCK-8 formazan products were measured at 460 nm using a microplate spectrophotometer (VERSA Max, Molecular Device Co., Sunnyvale, CA, USA).

### 2.4. Observation of Cellular and Nuclear Morphology

The cellular morphology was observed under an inverted microscope (Carl Zeiss, Oberkochen, Germany). As described previously [27], nuclear morphology was acquired using a fluorescence microscope (Carl Zeiss) by 4,6-diamidino-2-phenylindole (DAPI; Sigma-Aldrich Chemical Co.) staining.

### 2.5. Flow Cytometric Analysis for Cell Death and Cell Cycle Distribution

To estimate the mode of cell death, the cells were harvested and stained with FITC Annexin V Apoptosis Detection Kit (BD Biosciences, San Diego, CA, USA) for 20 min, according to the manufacturer’s protocol. The fluorescence intensity was detected using flow cytometry (BD Biosciences), and the annexin V-negative cells and annexin V-positive cells were considered to be necrotic and apoptotic, respectively [28]. To quantify the phase distribution of the cell cycle, the cells were stained with 40 μg/mL propidium iodine (PI; BD Biosciences) for 30 min and subjected to flow cytometry as previously described [29].

### 2.6. Analysis of Caspases Activities and Autophagy

Caspase-3, -8, and -9 enzyme-linked immunosorbent assay (ELISA) kits were purchased from R&D Systems Inc. (Minneapolis, MN, USA), and the activities were determined according to the manufacturer’s instruction. Autophagic vacuoles were analyzed using a Cyto-ID autophagy detection kit (Enzo Life Sciences Inc., FGA, NY, USA). DAPI was used to counterstain the nuclei. Stained cells were observed on a fluorescence microscope.

### 2.7. Western Blot Analysis

Total protein and mitochondrial protein were extracted by the PRO-PREP Protein Extraction Solution (Intron Biotechnology, Gyeonggi-do, Korea) and a mitochondria isolation kit (Thermo Fisher Scientific, Waltham, MA, USA), respectively. As previously described [30], equal proteins were separated by SDS-PAGE and transferred to a PVDF membrane, and subsequently, the membranes were probed specific primary antibodies (Supplementary Table S1). After incubation for overnight, the corresponding secondary antibodies were added and incubated. The protein expression was detected by a Fusion FX Image system (Vilber Lourmat, Torcy, France).

### 2.8. Intracellular ROS Detection

Cells were stained with 5,6-carboxy-2’,7’-dichlorodihydrofluorescein diacetate (DCF-DA; Invitrogen-Gibco) to assess intracellular ROS levels, and images were visualized by a fluorescence microscope [25].

### 2.9. Mitochondrial Membrane Potential (MMP, ∆Ψm) Analysis

Cells were stained with 5,5’,6,6’-tetrachloro-1,1’,3,3’-tetraethylimidacarbocyanine iodide (JC-1; Invitrogen-Gibco) to investigate the MMP (∆*Ψm*), and fluorescence images were captured by fluorescence microscope [29].

### 2.10. Immunofluorescence

To observe the expression of γH2AX, immunofluorescence staining was performed as described previously [31]. Information on the antibodies used was provided in Supplementary Table S1.

DAPI was used to counterstain the nuclei, and stained cells were observed by fluorescence microscope.

### 2.11. Statistical Analysis

The data are expressed as the mean ± SD. GraphPad Prism 5.03 (GraphPad Software Inc., La Jolla, CA, USA) software was used for statistical analyses. Significant differences were analyzed using the analysis of variance (ANOVA) followed by Tukey’s test. Probability values of *p* < 0.05 were considered to indicate significant.

## 3. Results

### 3.1. UPM Has Cytotoxicity in ARPE-19 Cells

**Figure 1:**
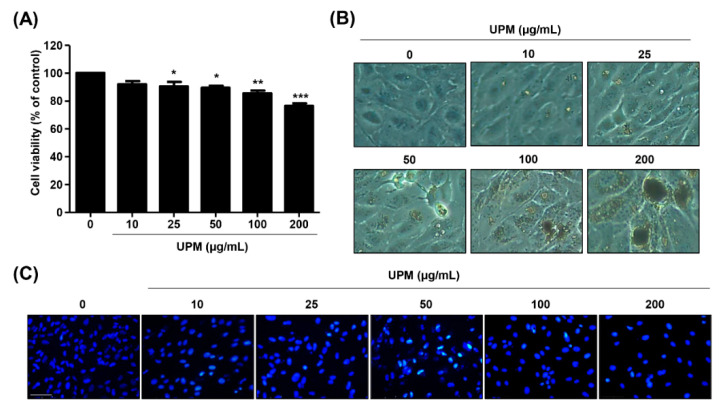
Urban particulate matter (UPM) induces slight cytotoxicity in ARPE-19 cells. ARPE-19 cells were incubated with UPM for 24 h. (**A**) Cytotoxicity was measured by a CCK-8 assay. Data are expressed as the mean ± SD (*n* = 6). * *p* < 0.05, ** *p* < 0.01, and *** *p* < 0.001 when compared to untreated cells. (**B**) Morphological cellular changes were observed under an inverted microscope. (**C**) DAPI staining was performed to visualize the morphological changes in the nucleus and was pictured under a fluorescence microscope. Scale bar: 75 μm.

To evaluate the effect of UPM on ARPE-19 cell viability, we performed a CCK-8 assay. Figure 1A shows that UPM significantly induced cytotoxicity in a dose-dependent manner. At 200 μg/mL UPM, the treated cells exhibited an approximately 76% viability compared with the untreated cells. Morphologically, we observed a marked accumulation of UPM around the cells that showed cell swelling, including an enlarged and flattened shape and the formation of cytoplasmic blebs (Figure 1B). Meanwhile, the DAPI staining results showed that although partial chromatin condensation was observed in UPM-stimulated cells, typical apoptotic DNA chromatin condensation was not exhibited (Figure 1C).

### 3.2. UPM Induces Necrosis and Autophagy but Not Apoptosis in ARPE-19 Cells

**Figure 2:**
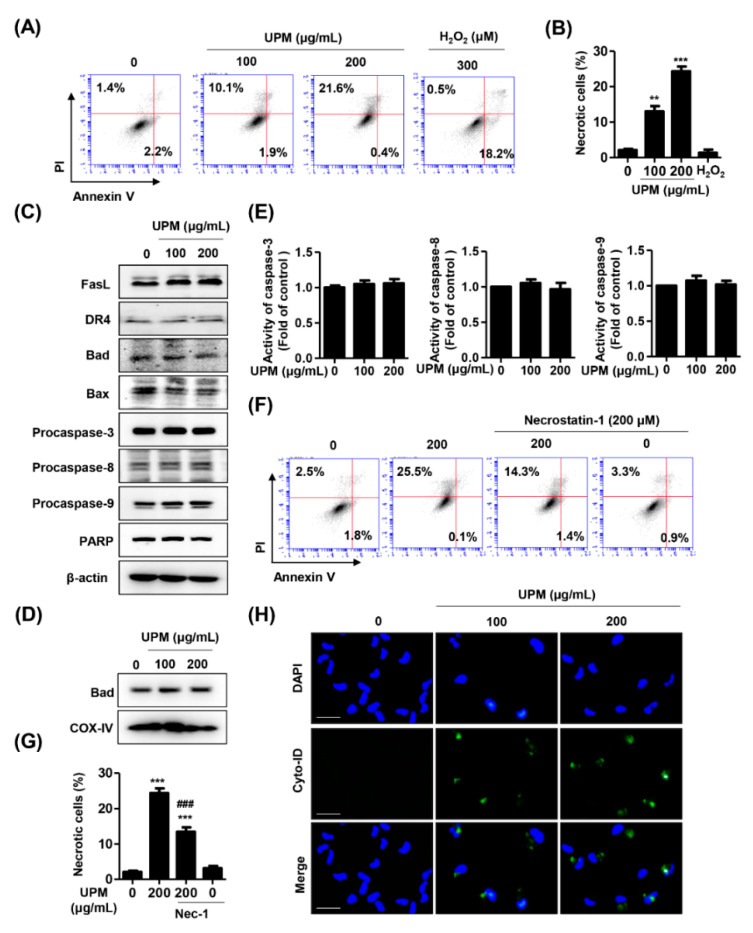
UPM induces necrosis and autophagy without apoptosis in ARPE-19 cells. (**A**–**E**) The cells were treated with the indicated concentration of UPM for 24 h. (**A**) The representative histograms of the cells stained with annexin V/propidium iodine (PI) and analyzed using a flow cytometer with necrotic cells identified as annexin V^−^/PI^+^ cells (upper left quadrant) and apoptotic cells defined as annexin V-positive cells (lower left quadrant). (**B**) The percentages of necrotic cells. (**C**) The expression of apoptosis-regulatory proteins. (**D**) The expression of Bad in mitochondrial fraction. (**E**) Relative activities of caspase-3, -8, and -9. (**F**) The representative histograms of ARPE-19 cells pretreated with 200 μM necrostatin-1 (Nec-1) for 1 h and incubated with 200 μg/mL UPM for 24 h and were stained with annexin V/PI. (**G**) The percentages of necrotic cells. Data are expressed as the mean ± SD (*n* = 5). ** *p* < 0.01 and *** *p* < 0.001 when compared to untreated cells. ^###^ *p* < 0.001 compared to 200 μg/mL UPM-treated cells. (**H**) Cyto-ID-stained cells (autophagic cells, green) and DAPI-stained cells (nuclei, blue) were captured under fluorescence microscopy. Scale bar: 75 μm.

To examine the cause of UPM-induced cytotoxicity with the lack of apoptotic chromatin condensation, we performed annexin V/PI staining, which is implicated in the identification of cell death modes. The results showed that the annexin V^−^/PI^+^ cell population, which was considered to be necrotic, was significantly increased by exposure to 200 μg/mL UPM (Figure 2A,B). However, the annexin V^+^/PI^+^ cell population that was considered to be apoptotic was not affected following UPM treatment. Meanwhile, apoptotic cell population was greatly increased in response to 300 μM H_2_O_2_ that was used as a positive control for apoptosis. In this respect, we considered that UPM-mediated cytotoxicity was involved in necrotic cell death but not apoptosis. To reconfirm whether UPM was involved in apoptosis in ARPE-19 cells, the expression of apoptosis-regulatory proteins was evaluated using Western blot analysis. Figure 2C shows that UPM did not affect the expression of death receptors and Bcl2 families and cleavage of poly (ADP-ribose) polymerase (PARP). Moreover, we confirmed that the expression of Bad was also unchanged by UPM exposure in mitochondrial fraction (Figure 2D). In addition, UPM did not affect both the expression of pro-caspases and activity of caspases (Figure 2C,E). Furthermore, with the pretreatment with necrostatin-1, an inhibitor of necrosis, approximately 50% suppressed the increase in UPM-induced necrotic cells (Figure 2F,G). This result suggested that UPM induced a cytotoxic effect that partially resulted from necrosis. Thus, we further investigated whether autophagy, another mode of cell death, is involved in UPM-mediated cytotoxicity. As a result, we found that Cyto-ID-stained cells were enhanced following exposure to UPM (Figure 2H). Based on these results, we considered that UPM induced necrotic and autophagic cell death, but no apoptosis in ARPE-19 cells.

### 3.3. UPM Promotes Cell Cycle Arrest at the G2/M Phase in ARPE-19 Cells

**Figure 3:**
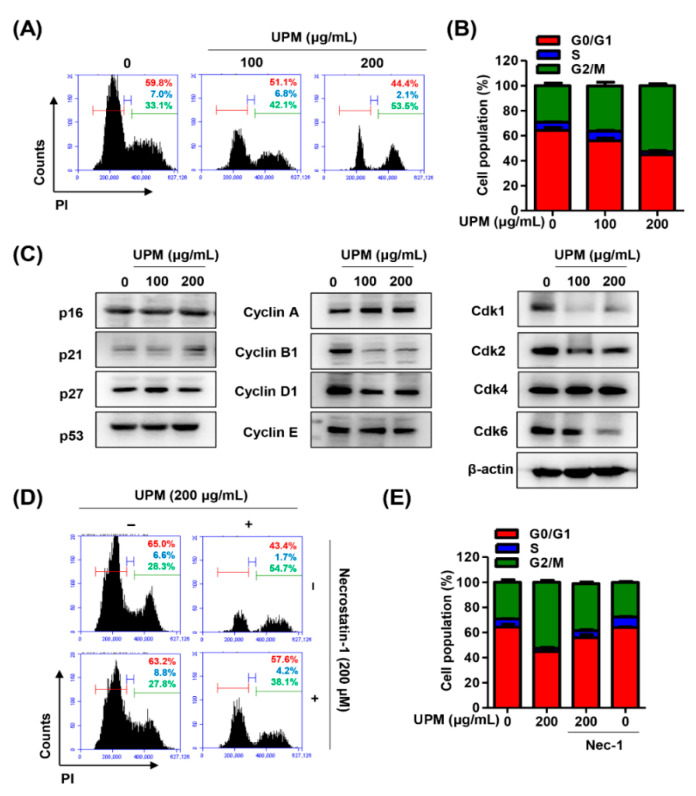
UPM mediates G2/M cell cycle arrest in ARPE-19 cells. (**A**) The representative histograms of ARPE-19 cells incubated with PI for flow cytometry analysis. (**B**) The average percentages of ARPE-19 cells incubated with PI in each phase of the cell, except for the cells at the sub-G1 phase. (**C**) The expression of cell cycle-regulatory proteins. (**D**) The representative histograms of ARPE-19 cells pretreated with 200 μM necrostatin-1 for 1 h and then treated with 200 μg/mL UPM for 24 h. The cells were stained with PI. (**E**) The average percentages of ARPE-19 cells pretreated with 200 μM necrostatin-1 for 1 h and treated with 200 μg/mL UPM for 24 h in each phase of the cell cycle, except for the cells at the sub-G1 phase.

Next, to evaluate whether UPM-induced cytotoxicity accompanied by necrosis and autophagy was due to a change in the cell cycle, alterations in cell cycle distribution were assessed. The flow cytometric analysis using PI staining indicated that UPM gradually induced a dose-dependent effect on G2/M phase arrest (Figure 3A,B and Supplementary Figure S1). At 200 μg/mL UPM exposure, the cells showed that the G2/M population increased from approximately 33% to 54% compared to the control. Additionally, at the molecular level, it was found that the expression of p21 was increased under stimulation with UPM, while the expression levels of p16, p27, and p53 were not changed (Figure 3C). Furthermore, other cell cycle regulators, including cyclin B1, cyclin D1, cyclin-dependent kinase (Cdk) 1, Cdk 2, and Cdk 6, were decreased. Meanwhile, UPM-mediated G2/M cell cycle arrest was partially attenuated under necrostatin-1 treatment (Figure 3D,E).

### 3.4. UPM Disrupts DNA and Mitochondria in ARPE-19 Cells

**Figure 4:**
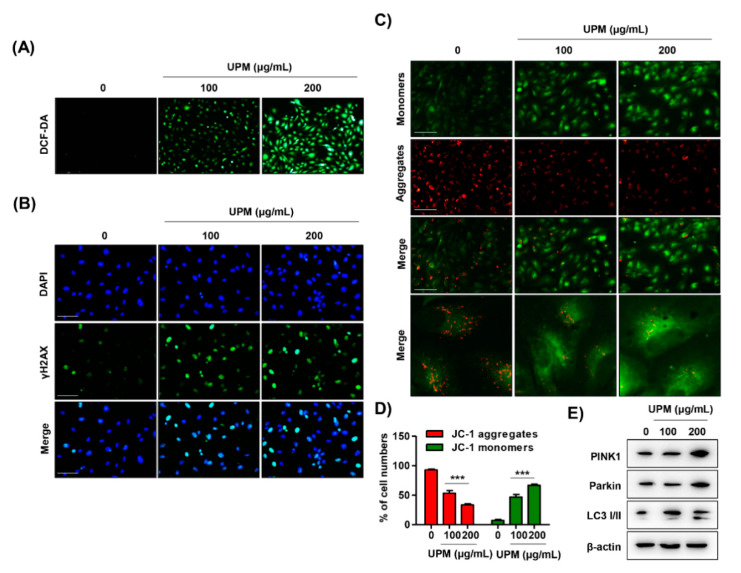
UPM triggers DNA and mitochondrial disorders in ARPE-19 cells. (**A**) Fluorescence images of ARPE-19 cells stained with 10 μM DCF-DA. Intracellular reactive oxygen species (ROS) generation was identified as a DCF-DA intensity that was observed under a fluorescence microscope. Scale bar: 200 μm. (**B**) Fluorescence images of the cells immunostained with γH2AX antibody (green) and visualized using a fluorescence microscope. DAPI was used to counterstain the nuclei (blue). Scale bar: 75 μm. (**C**) Fluorescence images of the cells stained with 10 μM JC-1 with the monomer showing green fluorescence defining low MMP (∆*Ψm*) and aggregates showing red fluorescence characterizing high MMP (∆*Ψm*). Scale bar: 75 μm. (**D**) Quantification of JC-1 red and green intensity. Data are expressed as the mean ± SD (*n* = 5). *** *p* < 0.001 when compared to untreated cells. (**E**) The expression of mitophagy-regulated proteins.

To identify whether ROS were involved in UPM-mediated cytotoxicity, we preferentially observed alterations in intracellular ROS levels and subsequently assessed their influence on organelles. As a result of DCF-DA staining, intracellular ROS levels were markedly enhanced by UPM exposure (Figure 4A and Supplementary Figure S1). Moreover, the fluorescence intensity of γH2AX, a DNA damage marker, was substantially increased in UPM-stimulated cells (Figure 4B). In addition, the exposure to UPM in a dose-dependent manner induced the loss of MMP (∆*Ψm*), which was accompanied by both the upregulation of the expression of JC-1 monomers and the downregulation of the expression of JC-1 aggregates (Figure 4C,D and Supplementary Figure S1). Furthermore, UPM promoted the expression of mitophagy regulators, including PTEN-induced kinase 1 (PINK1), Parkin, and light chain 3 (LC3) I/II (Figure 4E).

### 3.5. The Excess ROS Induced by UPM Triggers Necrosis, Autophagy, and Cell Cycle Arrest in ARPE-19 Cells

**Figure 5:**
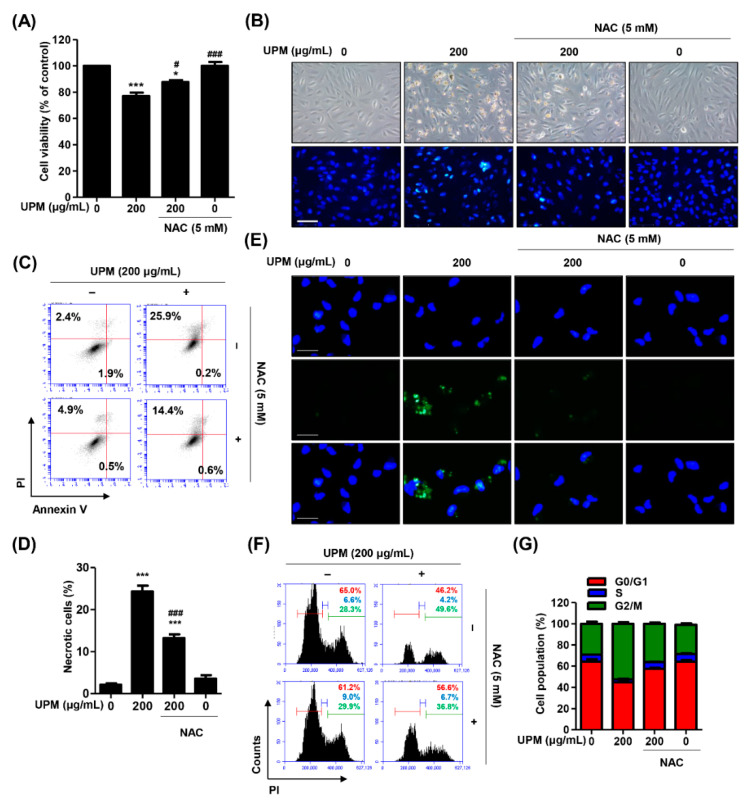
Suppression of ROS attenuates UPM-induced necrosis, auophagy, and cell cycle arrest in AREP-19 cells. The cells were pretreated with 5 mM NAC for 1 h and then treated with 200 μg/mL UPM for 24 h. (**A**) Cell viability measured by a CCK-8 assay. Data are expressed as the mean ± SD (*n* = 4). * *p* < 0.05 and *** *p* < 0.001 when compared to untreated cells. ^#^ *p* < 0.05 and ^###^ *p* < 0.001 compared to 200 μg/mL UPM-treated cells. (**B**) The shape of the cell and the morphological changes in the nucleus. Scale bar: 75 μm. (**C**) Representative histograms of cell death mode. (**F**) Cell cycle profiles derived from flow cytometry analysis. (**D**) The percentages of necrotic cells. Data are expressed as the mean ± SD (*n* = 5). *** *p* < 0.001 when compared to untreated cells. *** *p* < 0.001 compared to 200 μg/mL UPM-treated cells. (**E**) Cyto-ID-stained cells (autophagic cells, green) and DAPI-stained cells (nuclei, blue) captured under fluorescence microscopy. Scale bar: 75 μm. (**G**) The average percentages of cells in each phase of the cell cycle.

Based on the above results showing that UPM promoted intracellular ROS generation and organelle dysfunction, we investigated the role of ROS in UPM-induced necrosis and autophagy. The pretreatment with NAC, a potential ROS scavenger, significantly recovered UPM-induced cytotoxicity and morphological changes (Figure 5A,B). Additionally, Figure 5C,D shows that NAC greatly suppressed the increase in necrotic cells by UPM. Furthermore, NAC markedly suppressed the expression of Cyto-ID-positive cell population (Figure 5E). Likewise, the UPM-induced cell cycle arrest at the G2/M phase was also restored under NAC treatment (Figure 5F,G). These results suggested that UPM promoted intracellular ROS generation, which is involved in necrosis, autophagy, and cell cycle arrest.

### 3.6. ROS Directly Regulate UPM-Induced DNA and Mitochondrial Damage in ARPE-19 Cells

**Figure 6:**
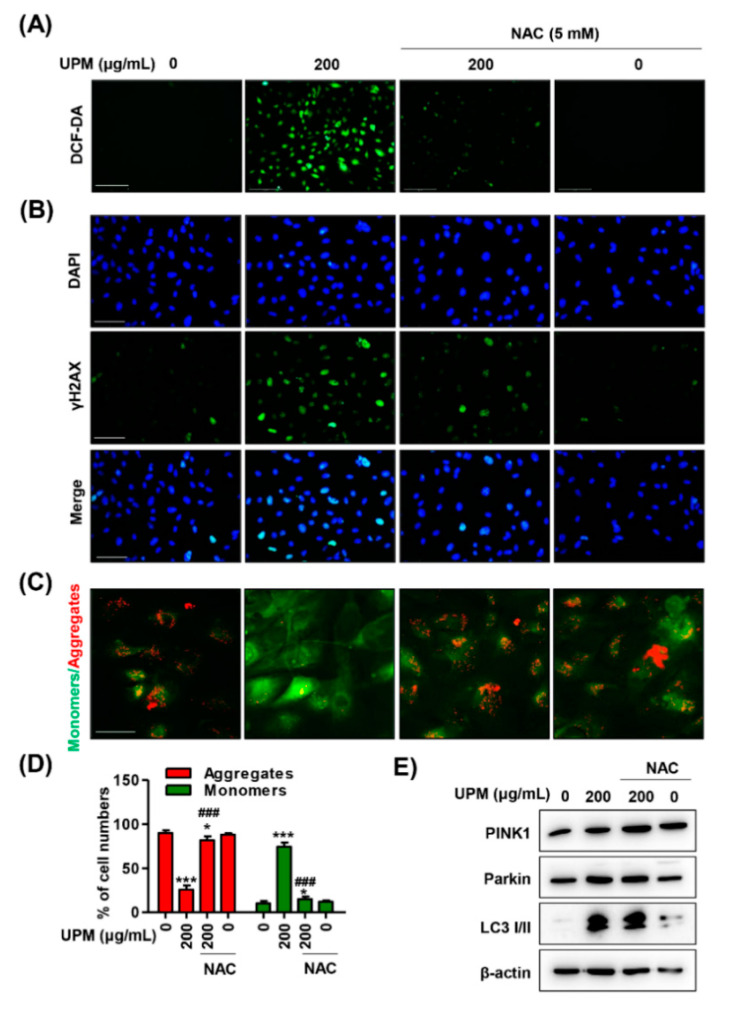
Inhibition of ROS suppresses UPM-induced DNA and mitochondrial damage in AREP-19 cells. (**A**) Representative fluorescence images of DCF-DA-stained cells. Scale bar: 200 μm. (**B**) The fluorescence expression of γH2AX (green). DAPI was used to counterstain the nuclei (blue). Scale bar: 75 μm. (**C**) Representative images of JC-1 fluorescence staining. Monomers showing green fluorescence define low MMP, and aggregates showing red fluorescence characterize high MMP. Scale bar: 25 μm. (**D**) Quantification of JC-1 red and green intensities. Data are expressed as the mean ± SD (*n* = 4). * *p* < 0.05 and *** *p* < 0.001 when compared to untreated cells. ^###^ *p* < 0.001 compared to 200 μg/mL UPM-treated cells. (**E**) The expression of mitophagy-regulated proteins.

Next, we verified whether UPM-induced ROS triggered organelle dysfunction, because UPM results in necrosis and autophagy through ROS generation. As shown in Figure 6A, NAC markedly attenuated intracellular ROS generation and MMP (∆*Ψm*) loss following UPM exposure. The quantified results also showed that inhibition of ROS by NAC significantly suppressed the intensity of JC-1 monomers and DCF-DA (Figure 6B,C). Furthermore, we observed that NAC markedly inhibited the fluorescence expression of γH2AX in UPM-stimulated cells (Figure 6D). However, the upregulation of mitophagy regulators by UPM was not changed by NAC treatment (Figure 6E).

## 4. Discussion

The retina, the back segment of the eye, functions in vision control and composed of 10 layers that consist of 8 specific cells [32]. In particular, the RPE, the outer layer of the retina, plays an essential role in the vision process by exchanging nutrients, metabolic end products, and signal molecules with neighboring cells [32]. Therefore, RPE cells are critical for retinal homeostasis, and the loss and dysfunction of RPE cells are causative of AMD, which is the leading cause of blindness in the elderly [33]. Several genetic and environmental risk factors for AMD lead to inflammation, anatomical changes, and dysfunction in the retina, which can eventually lead to loss of vision [34]. Recently, it has been reported that air pollutants are considered one of the environmental risk factors for AMD and are involved in ROS overload by oxidative stress, which can lead to RPE cell death [19,24].

Apoptosis, necrosis, and autophagy are three major types of cell death in response to oxidative stress [35]. Apoptosis can be executed by the extrinsic and intrinsic pathways, both of which activate effector caspases, leading to the cleavage or degradation of cellular substrates, including PARP and histones, ultimately leading to apoptotic cell death [36,37]. Morphologically, apoptosis is characterized by cytoplasmic shrinkage, chromatin condensation and fragmentation, and the maintenance of the plasma membrane [36]. In contrast, necrosis is an unregulated form of cell death and is mediated by receptor-interacting protein kinases (RIPKs) that are involved in necrosome formation, subsequently leading to mitochondrial fission and cell death [38]. During necrosis, mitochondrial fragmentation and mitochondrial permeability transition pore (mPTP) opening occur, which lead to MMP (∆*Ψm*) loss, ATP depletion and ROS release, although the signaling pathways prefacing these events are unknown [36,39]. The morphological features of necrosis include cell swelling, an increase in cell volume, the formation of cytoplasmic vacuoles and blebs, and the eventual disruption of the cell membrane [36]. Autophagy is a predominant cell survival process of homeostasis that involves degradation of damaged organelles through formation of autophagosome [40]. Accumulated evidences suggested that autophagy is impaired in the aged retina, which contributes to the RPE dysfunction [41,42]. Although the current paradigm is that apoptosis is a major mechanism for RPE cell death by oxidative stress, the role of apoptosis in the RPE death mechanism in retinal disorders is still controversial [36]. In this regard, we investigated which cell death mode is involved in RPE dysfunction following UPM exposure and identified the cell death mechanism. Our results showed that UPM induced cell death with morphological changes by necrosis, but typical apoptotic DNA chromatin condensation did not occur (Figure 1). Furthermore, the findings from the flow cytometric analysis and immunoblots indicated that UPM mediated cell death caused by necrosis and did not affect the expression of apoptotic regulators (Figure 2A–E). Additionally, based on the result showing that necrostatin-1, a RIP1 kinase inhibitor, markedly restored UPM-mediated necrotic cell death (Figure 2F,G), our findings demonstrated that UPM-induced cytotoxicity is involved in necrotic cell death in the RPE. This finding supports the previous results of Hanus et al. [43] showing that during RPE cell death upon oxidative stress induced by pro-oxidants, cardinal features of necrosis were observed but apoptosis features were not observed. The results also suggested that necrostatin-1 and the silencing of RIPK3 largely prevented oxidative stress-induced RPE death, which established that necrosis is a major type of RPE cell death in response to oxidative stress [43]. More recently, Kang et al. [16] demonstrated that long-term exposure to UPM led to anatomical changes in the retina whereas apoptosis did not occur. Although necrostain-1 significantly inhibited annexin V^−^/PI^+^ cells in the present study, approximately 50% suppressed the increase in UPM-induced necrotic cells by necrostain-1. This result suggested that UPM induces a cytotoxic effect that partially results from necrosis. We should keep in mind, however, that necrostain-1 could also partially influence apoptosis [44]. Importantly, we observed a dose dependence increased the expression of Cyto-ID, a specific dye for autophagic compartments [45], in UPM-treated ARPE-19 cells (Figure 2H). Several reports demonstrated that autophagy plays an essential role in bronchial cells and animals upon UPM exposure [46,47]. In a ocular system, a few studies reported that autophagy is involved in PM-mediated pathogenic mechanisms in corneal epithelial cells [48,49]. However, the role of autophagy on retina in response to UPM exposure has remained elusive. Based on these knowledge, our finding that UPM induced autophagy in ARPE-19 cells provides the first evidence of the effect of UPM on the posterior part of the eye. Taken together, our findings that UPM-induced RPE cell death is mainly caused by necrosis and autophagy can provide the explanation of a novel mechanism for AMD pathogenesis upon exposure to urban air pollution.

In response to stressful conditions, cells are able to block the cell cycle transiently or irreversibly [50]. In fact, the cellular response to DNA damage causes either cell cycle arrest or apoptosis [51]. Cell cycle progression is regulated by CDKs, and its activity is coordinated by binding of their essential regulatory subunits, cyclins [52]. Numerous studies have demonstrated that UPM may inhibit cell growth by cell death or reduction of proliferation, which has been linked to arrest in various steps of the cell cycle [53,54,55,56]. Zhang et al. [56] provided the evidence that PM_2.5_ induces cell cycle arrest in G1 phase in alveolar epithelial cells through the downregulation of cyclin E, A, and D1 and the upregulation of p21. Furthermore, Wu et al. [55] suggested that UPM leads to G2/M cell cycle arrest, DNA damage, and cell death in bronchial epithelial cells. In addition, Gualtieri et al. [53] reported that UPM causes mitotic arrest, resulting in cell death in bronchial epithelial cells. The same research team published the result that UPM induces a delay in G2 phase and augments ROS formation, which causes damage to the DNA and spindle apparatus [54]. Although accumulated evidence has demonstrated that UPM induces cell cycle arrest with cell death in various cell types, the cellular responses of RPE after exposure to UPM on cell cycle regulation are not known. In the present study, we found that UPM induced G2/M cell cycle arrest, along with the overexpression of γH2AX in the nucleus (Figure 3A,B and Figure 4B,C). Furthermore, our findings showed that UPM was attributed to modulation of cell cycle regulators, including the upregulation of p21 and the downregulation of cyclin B/D and CDK1/2/6, while the expression levels of p16, p27, and p53 were not altered (Figure 3C). p21 is known to act as a tumor suppressor and inhibits the activity of the cyclin/CDK complex, which in turn leads to cell cycle arrest [57]. The cell cycle arrest at the G2/M phase indicates that damage to intracellular DNA is difficult to repair [58]. One of the responses to DNA damage is the expression of γH2AX, which is an early sign of DNA damage induced by replication stalling [51]. The formation of γH2AX foci takes place immediately after the generation of DNA breaks, as well as replication stalling and single-stranded DNA breaks [51]. In bronchial epithelial cells, UPM promotes the breakage of DNA stands and triggers γH2AX activation in response to DNA damage, leading to cell death and mitotic arrest [53]. According to Fernandez-Capetillo et al., H2AX-null cells were defective in cell cycle progression at the G2/M phase after exposure to ionizing radiation [51]. Fragkos et al. [59] demonstrated that adeno-associated virus-induced DNA damage leads to γH2AX generation and subsequently results in cell cycle arrest by upregulating p21. On the other hand, in H2AX-deficient cells, the response to DNA damage causes apoptosis via p21 degradation, suggesting that H2AX is required for p21-induced cell cycle arrest [59]. Taken together, our results established that DNA damage by UPM-mediated cellular stress induces the expression of γH2AX and subsequently inhibits cell cycle progression through the upregulation of p21 in RPE cells. In addition, a RIP1 kinase inhibitor markedly restored UPM-induced cell arrest at G2/M phase (Figure 3D,E), suggesting that UPM-induced cell cycle arrest is involved in necrosis in RPE cells.

Accumulated evidence has consistently shown that ROS induced by oxidative stress are crucial mediators of UPM toxicity [60]. UPM contains redox-active components, such as heterocyclic polycyclic aromatic hydrocarbons (PAHs), nitro-PAHs, and various metals, which can lead to intracellular ROS generation by catalyzing Fenton’s reaction [61,62]. Given that mitochondria are a major cellular source of ROS, growing evidence suggests that it is imperative to understand the effect of PM on mitochondrial structure and function [63,64]. In this regard, it has been reported that oxidative stress and mitochondrial dysfunction occur in various cell types upon exposure to UPM [64,65]. Miao et al. [64] suggested that PM induces endothelial toxicity through alterations in mitochondrial morphology and function, including MMP (∆*Ψm*) loss and mPTP opening. The findings from Bhargava et al. [65] showed that PM exposure promotes ROS generation and results in mitochondrial membrane depolarization and alteration of mitochondrial respiratory chain enzyme activity in peripheral blood lymphocytes. Among the studies on the eye, Cui et al. [66] suggested that PM causes a delay in corneal epithelium wound healing and ROS formation might play a critical role in this process. Recently, Somayajulu et al. [67] established that PM_2.5_ triggers ROS, which results in increased mRNA levels of oxidative stress and inflammation in mouse and human corneal epithelial cells. Although a few studies have suggested that PM triggers ROS generation in corneal and conjunctival epithelium, no studies on ROS and mitochondrial function upon PM have been reported. Furthermore, no studies have focused on the role of ROS in the retina, except for our previously published report, in which we demonstrated that diesel PM_2.5_ promotes intracellular ROS generation, which causes mitochondrial dysfunction, including suppression of mitochondrial activity and loss of MMP (∆*Ψm*), subsequently leading to retinal dysfunction [25]. In the present study, we found that UPM promoted intracellular ROS generation and concurrently induced MMP (∆*Ψm*) loss in RPE cells (Figure 4A,D,E). Notably, blocking ROS generation by NAC partially reversed UPM-mediated cytotoxicity, necrosis, and G2/M cell cycle arrest, as well as almost totally suppressed UPM-induced autophagy, DNA damage, and MMP (∆*Ψm*) loss (Figure 5 and Figure 6). These results demonstrated that UPM-induced RPE cell death is caused by necrosis and autophagy mechanisms that involve DNA damage-triggered cell cycle arrest and mitochondrial damage-triggered MMP (∆*Ψm*) loss and that are partially regulated by ROS generation.

Mitophagy is a homeostatic process for the selective degradation of dysfunctional and damaged mitochondria [68]. Under pathological conditions, active PINK1 accumulates on the outer mitochondrial membrane (OMM) to promote Parkin recruitment, and subsequently, Parkin triggers the polyubiquitination of several OMM proteins [69]. In turn, several adaptor molecules bind to polyubiquitinated proteins and initiate the formation of autophagosomes by binding with LC3 [69]. Recently, it was established that PM_2.5_ induces mitochondrial dynamics in liver fibroblasts and may trigger mitophagy via the PINK1/Parkin signaling pathway by ROS generation [70]. Another study reported that the ambient pollutant acrolein results in mitochondrial DNA damage through mitochondrial fission and mitophagy in lung epithelial cells and fibroblasts [71]. According to Wang et al.’s finding, the dysfunction of mitochondrial degradation by mitophagy can cause the activation of the mitochondrial-dependent apoptosis pathway [71]. In the present study, we found that UPM promoted the expression of mitophagy regulators, including PINK1, Parkin, and LC3 I/II, but this upregulation by UPM was not affected by NAC (Figure 4F and Figure 6E). Based on this result, UPM induced mitochondrial dysfunction via intracellular ROS generation in the RPE, while mitophagy was regulated by a ROS-independent pathway. Although further studies on the regulatory mechanisms of ROS and mitophagy in UPM-exposed RPE are required, our findings provide the first evidence that UPM induces mitochondrial dysfunction and leads to mitophagy in RPE.

## 5. Conclusions

**Figure 7:**
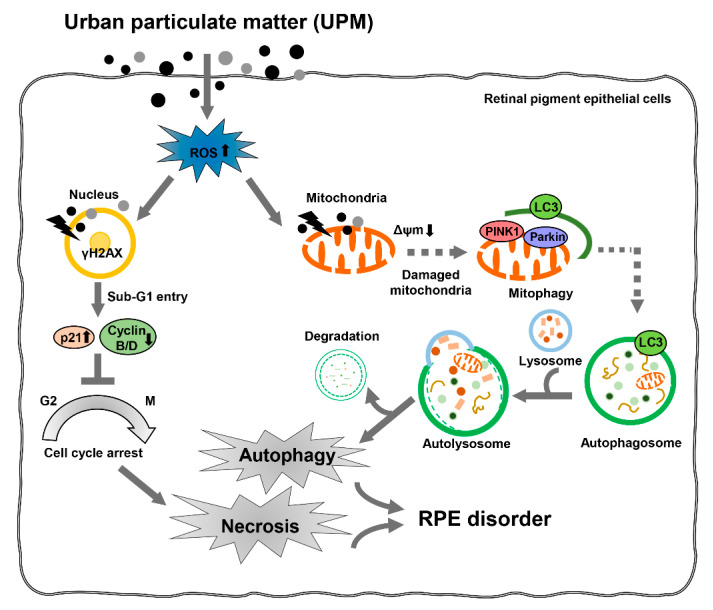
UPM promotes necrosis and autophagy via ROS-mediated cellular disorders that are accompanied by cell cycle arrest and mitophagy in ARPE-19 cells. UPM induces cytotoxicity, which is due to autophagic and necrotic cell death with cell cycle arrest at the G2/M phase. Furthermore, UPM markedly enhances DNA damage with the overexpression of γH2AX. In addition, UPM notably increases mitochondrial dysfunction with MMP (∆*Ψm*) loss and induces mitophagy. This UPM-mediated cellular damage is attributed to intracellular ROS production but not mitophagy. In conclusion, ROS-mediated cellular damage with autophagy and necrosis may be the critical mechanisms underlying UPM-induced retinal disorders.

Overall, our results showed that UPM promotes RPE cell death, involving necrosis and autophagy but not apoptosis. Furthermore, exposure to UPM leads to cell cycle arrest at the G2/M phase, which may be caused by the formation of γH2AX foci and subsequent p21 upregulation. In addition, UPM causes mitochondrial dysfunction with MMP (∆*Ψm*) loss and upregulates mitophagy. These UPM-mediated cellular alterations are regulated by ROS production, except for mitophagy. Although further studies are needed to identify the role of mitophagy in UPM-induced RPE injury, the present study provides the first evidence that ROS-mediated cellular damage with necrosis and autophagy may be the critical mechanism of UPM-induced retinal disorders (Figure 7). Taken together, our findings suggested that targeting oxidative stress-induced necrosis and autophagy in the RPE may be a viable approach to preventing urban air pollutant-induced retinal disorders, including AMD.

## Data Availability

The data presented in this study are available within the article and its supplementary material. Other data that support the findings of this study are available upon request from the corresponding authors.

## Supplementary Materials

The following are available online at https://www.mdpi.com/2076-3921/10/2/149/s1, Figure S1: Low concentration of UPM induced cellular dysfunction in ARPE-19 cells., Table S1: Primary and secondary antibodies used for immunoblotting and immunofluorescence.
