# Supplementary material for: Urban Aerosol Particulate Matter Promotes Necrosis and Autophagy via Reactive Oxygen Species-Mediated Cellular Disorders that Are Accompanied by Cell Cycle Arrest in Retinal Pigment Epithelial Cells
Source: Antioxidants (Basel). 2021 Jan 20;10(2):149. doi: 10.3390/antiox10020149 (PMC7909535; doi:10.3390/antiox10020149)
Supplement: Supplementary file 1 [file antioxidants-10-00149-s001.pdf]

Supplementary Table 1. Primary and secondary antibodies used for immunoblotting and immunofluorescence.

Antibodies	Supplier	Item No.	Dilution
β-actin	Santa Cruz Biotechnology, Inc. (Santa Cruz, CA, USA)	sc-1615	1:2,000
Bad	Santa Cruz Biotechnology, Inc.	sc-8044	1:500
Bax	Santa Cruz Biotechnology, Inc.	sc-4780	1:500
Caspase-3	Santa Cruz Biotechnology, Inc.	sc-7272	1:1,000
Caspase-8	Santa Cruz Biotechnology, Inc.	sc-56070	1:1,000
Caspase-9	Santa Cruz Biotechnology, Inc.	sc-7885	1:1,000
Cdk1	Abcam Inc. (Cambridge, UK)	ab131450	1:500
Cdk2	Santa Cruz Biotechnology, Inc.	sc-6248	1:1,000
Cdk4	Santa Cruz Biotechnology, Inc.	sc-23896	1:1,000
Cdk6	Santa Cruz Biotechnology, Inc.	sc-7961	1:1,000
COX-IV	Santa Cruz Biotechnology, Inc.	sc-376731	1:1,000
Cyclin A	Santa Cruz Biotechnology, Inc.	sc-239	1:1,000
Cyclin B	Santa Cruz Biotechnology, Inc.	sc-245	1:1,000
Cyclin D	Santa Cruz Biotechnology, Inc.	sc-8396	1:1,000
Cyclin E	Santa Cruz Biotechnology, Inc.	sc-377100	1:1,000
DR4	Santa Cruz Biotechnology, Inc.	sc-8411	1:1,000
FasL	Santa Cruz Biotechnology, Inc.	sc-957	1:1,000
γH2AX	Cell Signaling Technology (Beverly, MA, USA)	#9718	1:100
LC3 I/II	Cell Signaling Technology	#12741	1:1,000
p16	Santa Cruz Biotechnology, Inc.	sc-56330	1:1,000
p21	Cell Signaling Technology	#2947	1:1,000
p27	Cell Signaling Technology	#3686	1:1,000
p53	Cell Signaling Technology	#2524	1:1,000
Parkin	Cell Signaling Technology	#4211	1:1,000
PARP	Santa Cruz Biotechnology, Inc.	sc-8007	1:1,000
Pink1	Cell Signaling Technology	#6946	1:1,000
goat anti-mouse IgG-HRP	Santa Cruz Biotechnology, Inc.	sc-2005	1:1,500
goat anti-rabbit IgG-HRP	Santa Cruz Biotechnology, Inc.	sc-2004	1:1,500
Alexa Fluor 488-labeled goat anti-rabbit IgG	Thermo Fisher Scientific, Waltham, MA, USA	A32731	1:200

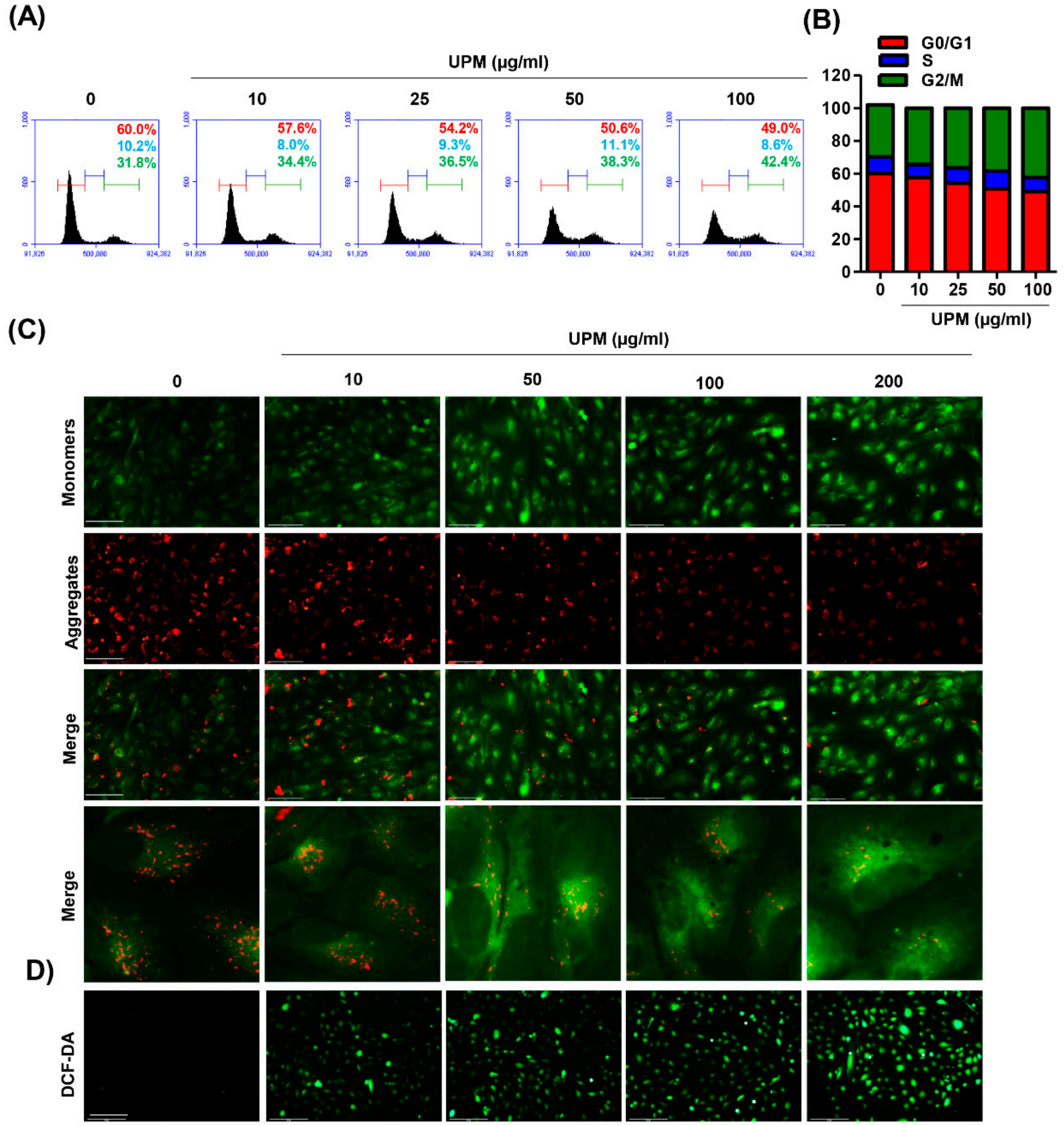

Supplementary Figure S1. Low concentration of UPM induced cellular dysfunction in ARPE-19 cells. (A and B) ARPE-19 cells were incubated with PI for flow cytometry analysis. (A) Representative histograms. (B) The average percentages of cells in each phase of the cell cycle are displayed, except for the cells of sub-G1 phase. (C) Monomer showing green fluorescence defining low MMP (ΔΨm) and aggregates showing red fluorescence characterizing high MMP (ΔΨm). Representative fluorescence images. Scale bar; 75 μm. (D) Intracellular ROS generation was identified as DCF-DA intensity that was observed under a fluorescence microscope. Scale bar; 200 μm.
